# *Trans*‐3,5,4´‐trimethoxystilbene reduced gefitinib resistance in NSCLCs via suppressing MAPK/Akt/Bcl‐2 pathway by upregulation of miR‐345 and miR‐498

**DOI:** 10.1111/jcmm.14086

**Published:** 2019-01-30

**Authors:** Min Lu, Bin Liu, Hui Xiong, Fang Wu, Chunhong Hu, Ping Liu

**Affiliations:** ^1^ Department of Oncology The Second Xiangya Hospital Central South University Changsha Hunan China

**Keywords:** apoptosis, gefitinib resistance, miR‐345 and miR‐498, NSCLC, TMS

## Abstract

Despite initial dramatic efficacy of epidermal growth factor receptor (EGFR) tyrosine kinase inhibitors (EGFR‐TKIs) in EGFR‐mutant lung cancer patients, subsequent emergence of acquired resistance is almost inevitable. Resveratrol and its derivatives have been found to exert some effects on EGFR‐TKI resistance in non‐small cell lung cancer (NSCLC), but the underlying mechanisms remain unclear. We screened several NSCLC cell lines with gefitinib resistance by MTT assay and analysed the miR‐345/miR‐498 expression levels. NSCLC cells were pre‐treated with a resveratrol derivative, *trans*‐3,5,4‐trimethoxystilbene (TMS) and subsequently challenged with gefitinib treatment. The changes in apoptosis and miR‐345/miR‐498 expression were analysed by flow cytometry and q‐PCR respectively. The functions of miR‐345/miR‐498 were verified by CCK‐8 assay, cell cycle analysis, dual‐luciferase reporter gene assay and immunoblotting analysis. Our results showed that the expression of miR‐345 and miR‐498 significantly decreased in gefitinib resistant NSCLC cells. TMS pre‐treatment significantly upregulated the expression of miR‐345 and miR‐498 increasing the sensitivity of NSCLC cells to gefitinib and inducing apoptosis. MiR‐345 and miR‐498 were verified to inhibit proliferation by cell cycle arrest and regulate the MAPK/c‐Fos and AKT/Bcl‐2 signalling pathways by directly targeting MAPK1 and PIK3R1 respectively. The combination of TMS and gefitinib promoted apoptosis also by miR‐345 and miR‐498 targeting the MAPK/c‐Fos and AKT/Bcl‐2 signalling pathways. Our study demonstrated that TMS reduced gefitinib resistance in NSCLCs via suppression of the MAPK/Akt/Bcl‐2 pathway by upregulation of miR‐345/498. These findings would lay the theoretical basis for the future study of TMS for the treatment of EGFR‐TKI resistance in NSCLCs.

## 1 INTRODUCTION

Lung cancer is one of the most common malignancies, of which about 80% are non‐small cell lung cancer (NSCLC). Surgery is an effective method to cure NSCLC, but only about 30% of NSCLC patients have a chance of surgery. The remission rates of radiotherapy and chemotherapy for the treatment of NSCLC disease were only 25%‐35% and 15%‐20% respectively.^1^ In recent years, the study of targeting the epidermal growth factor receptor tyrosine kinase inhibitor (EGFR‐TKI) has become a hot spot in NSCLC treatment.^2^, ^3^, ^4^, ^5^ That is, by competitively binding extracellular ligand binding sites with ATP or other substrates, blocking EGFR tyrosine autophosphorylation and tyrosine kinase activation, leading to EGFR activation inhibition and downstream signal transduction disorder, ultimately inhibiting cell cycle progression, accelerating apoptosis, disrupting angiogenesis, invasion and metastasis.^2^, ^3^ Gefitinib is a commonly used EGFR‐TKI drug for NSCLC treatment. EGFR‐TKIs can prolong patients’ progression free survival and overall survival in NSCLC clinical treatment, especially in relatively sensitive patients, both in one‐line and multi‐line treatment and the side effects are mild.^4^ However, there is no significant effect of EGFR‐TKs on some patients, EGFR mutations for example; or patients initially sensitive to EGFR‐TKIs with good efficacy for some time (about 1‐1.5 years), but subsequently prone to develop disease progression and EGFR‐TKI resistance, the so‐called secondary resistance.^5^

*Trans*‐3,5,4′‐trimethoxystilbene (TMS), a polyphenolic compound known by the chemical name of Stilbene, is a synthetic analogues of resveratrol, a constituent of red wine, vegetables and Chinese medicines, *Polygonum cuspidatum* for example.^6^ TMS has been reported to possess more potent anticancer and antiangiogenic activities than resveratrol.^7^, ^8^, ^9^, ^10^ Resveratrol and its derivatives have been found to exert some effects on NSCLC,^11^ especially on EGFR‐TKI resistance, but the underlying mechanisms remain unclear.

MiRNAs are a group of non‐coding small RNAs about 22‐26 nucleotides, involved in the regulation of gene expression at the post‐transcriptional level. In recent years, miRNAs have become the focus of oncology research. Although only about 1% of human genes, miRNAs regulate about 30% of the human‐encoded protein genes involved in the occurrence and development of many tumours, including lung cancer.^12^, ^13^ Recent research found that miRNAs involved in a variety of tumour drug resistance, especially in NSCLC, can affect the chemosensitivity of gefitinib and other drugs involved in EGFR‐TKIs resistance.^14^, ^15^ MiR‐345 and miR‐498 were found to be downregulated in NSCLC patients and cell lines and closely associated with the tumour progression and poor prognosis,^16^, ^17^ but there were few reports about the molecular regulation mechanism of miR‐345 and miR‐498 in NSCLC, especially in the EGFR‐TKI resistance.

In this study, we have identified a remarkable sensitization to gefitinib and the anticancer effects of TMS by miR‐345/miR‐498 and their downstream targeted signalling pathways in NSCLC providing a better understanding of the biological activities and functions of TMS. Our findings provide new evidence for TMS as an effective complementary medicine for combination treatment with EGFR‐TKI in NSCLC.

## 2 MATERIALS AND METHODS

### 2.1 Cell culture and drug treatment

The human NSCLC cell lines PC‐9, H1975, A549, H1299 and PC‐9/GR were obtained from ATCC (US) and cultured in RPMI1640 medium supplemented with 10% v/v FBS (Gibco, USA) in a humidified atmosphere of 95% air and 5% CO_2_. To screen the gefitinib resistant cell strains, a dose gradient (0, 5, 10, 50, 100, 200, 500 μmol/L) of gefitinib (Sigma, USA) was administered for 48 hours. The gefitinib‐acquired resistant cell subline PC‐9/GR was established by chronic exposure of PC‐9 cells to medium with increasing concentrations of gefitinib as described previously.^18^ To confirm the best fit for TMS (Sigma) treatment, a certain concentration range (0, 0.5, 5, 50, 500 μmol/L) was administered for 24, 48 or 72 hours. After treatment with TMS and/or gefitinib, cells were collected for analysis.

### 2.2 MiRNA transfection

Human miRNA mimics/inhibitors and the corresponding negative controls (NC) were designed and synthesized by GenePharma (Shanghai, China). When the cells reached 80% confluence, the RNA oligonucleotides were transfected by Lipofectamine 3000 (Invitrogen, USA) according to the manufacturer's instructions.

### 2.3 3‐(4,5‐Dimethylthiazol‐2‐yl)‐2,5‐diphenyltetrazolium bromide assays

H1299 and PC‐9/GR cells were seeded in 96‐well plates at a concentration of 1 × 10^6^ cells/well in 100 μL RPMI1640 medium without FBS. Drugs in 1% DMSO were added to the cells at various concentrations and incubated for 24 hours. The controls were treated with 1% DMSO alone. 3‐(4,5‐Dimethylthiazol‐2‐yl)‐2,5‐diphenyltetrazolium bromide (MTT) solution (10 μL; 5 mg/mL, PBS) was added to each well for an additional incubation of 4 hours at 37°C. After the addition of 100 μL DMSO, the reaction solution was placed in the dark for 30 minutes to dissolve the blue formazan crystals. The absorbance at 570 nm was measured with a Multiscan Spectrum. The cell viability was calculated relative to the untreated group using the formula: cell viability (%) = [(ATreatment − Ablank)/(AControl − Ablank)] × 100%.

### 2.4 Flow cytometric analysis

The apoptosis analysis was performed with a fluorescein isothiocyanate (FITC)‐labelled Annexin V Apoptosis Detection Kit (Invitrogen) according to the manufacturer's instructions. Briefly, cells were harvested and concentrated to 1 × 10^5^ cells/mL. Five microlitres of FITC‐conjugated Annexin V and 5 μL of PI solution were added to 0.1 mL of sample solution following incubation in the dark for 30 minutes. Then, the samples were measured by a flow cytometer (FACSCanto II; BD Biosciences, USA) and the data were analysed using a FlowJo software (LLC, USA).

For cell cycle analysis, cells were collected, fixed and then stained with 50 μg/mL propidium iodide solution (Invitrogen). After 30 minute incubation, the samples were analysed by the BD flow cytometer and FlowJo software.

### 2.5 Quantitative real time‐polymerase chain reaction (qRT‐PCR)

Total RNA was prepared using TRIzol reagent (Invitrogen) following the manufacturer's instructions. Four micrograms of total RNA was used as a template to synthesize cDNA by a first strand cDNA kit (Takara, Japan). Quantitative real time‐polymerase chain reaction (qRT‐PCR) amplification was performed with a SYBR Green PCR kit (Takara) and analysis by a 7500 Thermocycler (Applied Biosystems, USA), according to the manufacturer's protocols. The relative differences in gene expression levels were calculated through a 2^−ΔΔCt^ method.

### 2.6 Immunoblotting analysis

Protein samples were prepared using M‐PER (Thermo Scientific, USA) and separated by sodium dodecyl sulfate polyacrylamide gel electrophoresis (SDS‐PAGE). After being transferred onto a PVDF membrane using the BioRad Mini Protean electrotransfer system (Bio‐Rad, USA), the membrane was blocked in 5% BSA solution for 1 hour at room temperature following incubation with primary antibodies at 4°C overnight. Then the membranes were washed by the Tris Buffered Saline Tween (TBST) 3‐5 times and 5 minutes each time and subsequently incubated with HRP‐conjugated secondary antibodies for another 1 hour at room temperature. Finally the immunoreactive bands were developed using RapidStep™ ECL Reagent (EMD Millipore) and visualized by a chemiluminescence system (Bio‐Rad). All primary antibodies were purchased from Abcam and used in dilution as recommended. Relative expression levels of proteins were quantified by integrated gray values of the bands normalized with the internal reference *β*‐actin.

### 2.7 Vector construction and dual luciferase reporter assay

The 3′‐UTR sequences of targeted genes containing the predicted interaction sites and the corresponding mutant sites were amplified by PCR using human cDNA template and recombined downstream of the *Firefly* luciferase gene of pMIR‐Reporter vector (Ambion, USA).

For dual‐luciferase reporter assays, HEK‐293T cells cultured to 80% confluence were co‐transfected with these recombined reporter constructs and miRNA mimics/inhibitors or NCs using Lipofectamine 3000. A plasmid pRL‐TK carrying the *Renilla* luciferase gene was also transfected to cells of every group to control for transfection efficiency. At 24 hours after transfection, cells were harvested and measured for luciferase activity using the Dual‐Luciferase^®^ Reporter (DLR™) Assay System (Promega, USA) following the manufacturer's instructions. The relative luciferase activity was determined by normalization of *firefly* luciferase activity or *Renilla* luciferase activity.

### 2.8 Subcutaneous xenograft construction

The research protocol was approved and conducted in accordance with the institutional ethical guidelines from the Institutional Animal Care and Use Committee of Xiangya Hospital affiliated to Central South University.

Four‐ to six‐week‐old BALB/c athymic mice were injected subcutaneously into the right flank with 10^7^ H1299 cells. When palpable tumours (approximately 75 mm^3^ in diameter) developed, the mice were randomly divided into seven groups: (1) Control, left untreated; (2) Gefitinib, 25 mg/kg daily orally by gavage; (3) TMS, 30 mg/kg daily orally by gavage; (4) Gefitinib+TMS, oral administration of both the drugs in the indicated dose; (5) Gefitinib+TMS+Antagomir‐NC; (6) Gefitinib+TMS+Antagomir‐345; (7) Gefitinib+TMS+Antagomir‐498. For the latter three groups, 1 nmol corresponding antogomiRNA oligonucleotides were additionally injected into the tumours before oral administration on the first day. The injections were given every 4 days and for four times in total. Each treatment group consisted of at least five mice. Tumour volume was calculated using the formula π/6 × larger diameter × (smaller diameter)^2^ as described previously.^19^ About a month later after administration, all the mice were killed and the tumour samples were collected for further analysis and detection.

### 2.9 Statistical analysis

Data are indicated as mean ± SD for at least three different determinations. Statistical analysis was performed with the SPSS software (Chicago, IL, USA). Differences between variants were analysed by the Student's *t* test or one‐way ANOVA. Date were regarded as statistically significant when *P* value was at least less than 0.05.

## 3 RESULTS

### 3.1 Lower expression of miR‐345 and miR‐498 in gefitinib resistant NSCLC cell lines than gefitinib sensitive NSCLC cell lines

**Figure 1:**
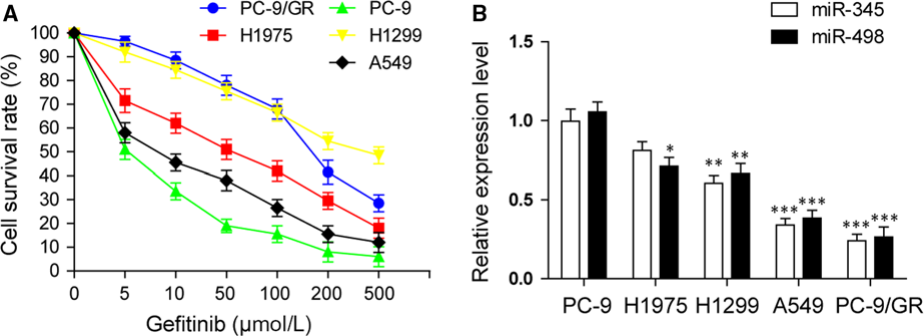
Lower expression of miR‐345 and miR‐498 in gefitinib resistant NSCLC cell lines than gefitinib sensitive NSCLC cell lines. (A) Cell viability analysis by MTT assay in NSCLC cell lines treated with a dose gradient of gefitinib for 48 hr. (B) qRT‐PCR analysis of miR‐345 and miR‐498 expression levels in NSCLC cell lines with different gefitinib resistance. All data are illustrated as mean ± SD and the asterisks show significant difference as *P < 0.05, **P < 0.01, ***P < 0.001 compared with the PC‐9 group

Non‐small cell lung cancer cells from different sources have different tolerance to gefitinib.^20^ We demonstrated the effects of gefitinib treatment with a certain concentration range (0, 5, 10, 50, 100, 200, 500 μmol/L) on cell viability of PC‐9, H1975, A549, H1299 and PC‐9/GR by the MTT assay (Figure 1A). The results revealed that the most gefitinib sensitive cells were PC‐9 and A549, H1975 were mildly resistant to gefitinib, whereas H1299 was much resistant to gefitinib. The PC‐9/GR was an induced‐specific gefitinib resistant strain. To confirm the correlation between miRNAs and tolerance of NSCLC to gefitinib, the expression levels of miR‐345 and miR‐498 in these cell lines were detected by qRT‐PCR (Figure 1B). Results suggested that the levels of miR‐345 and miR‐498, from highest to lowest were in PC‐9, H1975, H1299, A549 and PC‐9/GR, basically inverse correlating with gefitinib resistance.

### 3.2 TMS decreased the resistance of H1299 and PC‐9/GR cells to gefitinib by upregulating miR‐345 and miR‐498 in vitro and in vivo

**Figure 2:**
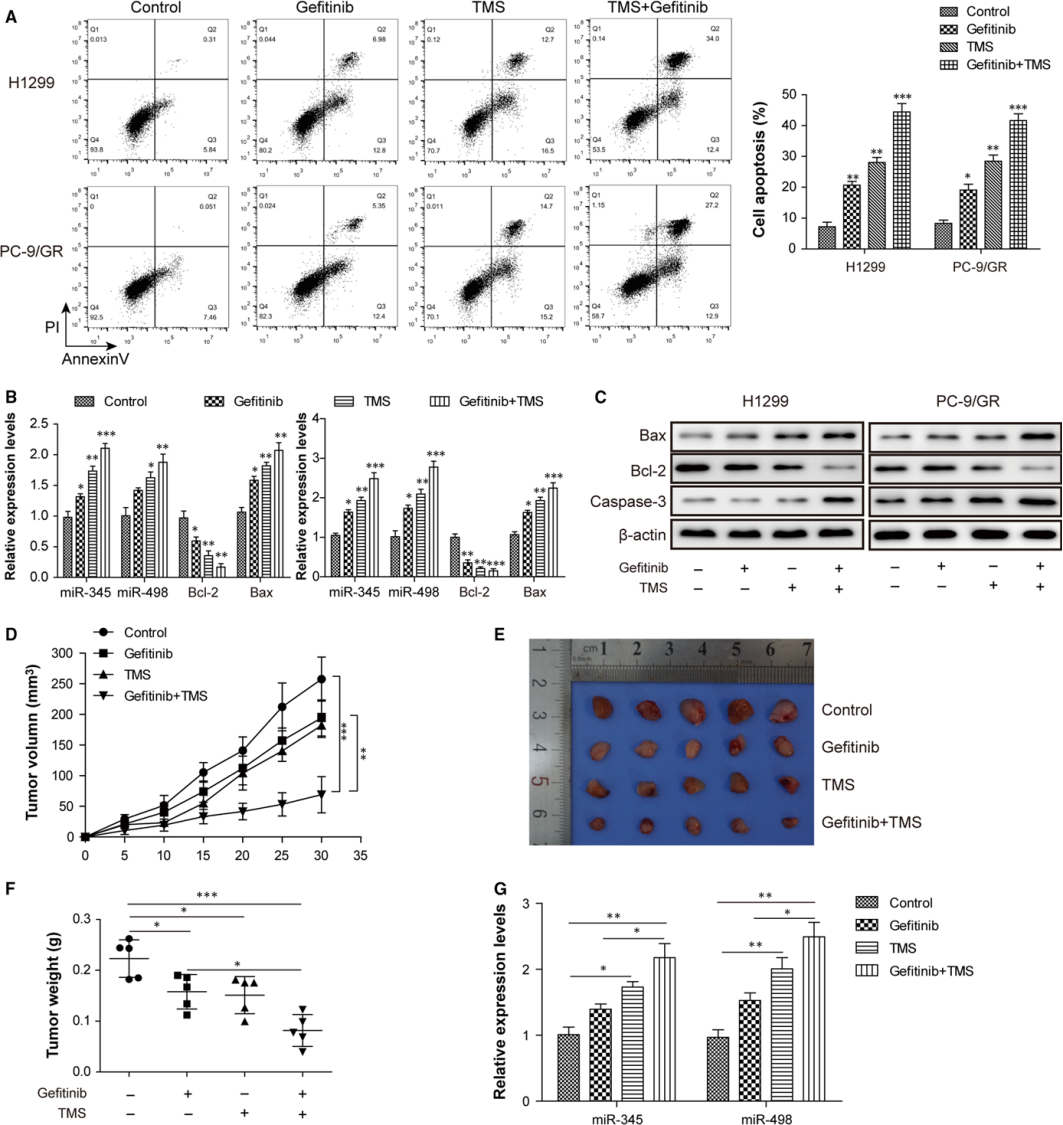
TMS decreased the resistance of H1299 and PC‐9/GR cells to gefitinib by upregulating miR‐345 and miR‐498 in vitro *and* in vivo. (A) Cell apoptosis analysis by Annexin‐V/PE and flow cytometry in H1299 and PC‐9/GR cells treated with or without 10 μmol/L gefitinib and 5 μmol/L TMS for 48 hr. Statistical analysis of the apoptotic positive rate is displayed in the histogram. (B) qRT‐PCR analysis of the expressions of miR‐345/miR‐498 and apoptosis marker genes Bcl‐2/Bax in H1299 and PC‐9/GR cells treated with or without gefitinib/TMS. (C) Immunoblotting analysis of apoptosis marker proteins Bcl‐2, Bax and Caspase‐3 expression in H1299 and PC‐9/GR cells treated with or without gefitinib/TMS. (D) Tumour volume growth delay curves for H1299 cell xenografts treated without or with gefitinib, TMS or the combination of the two by daily oral administration for 30 days. (E) The images of H1299 cell xenograft tumours from athymic mice of each group (n = 5) after 30‐day‐treatment for gefitinib/TMS. (F) The tumour weights of H1299 cell xenografts as described in E. (G) qRT‐PCR analysis of the expressions of miR‐345 and miR‐498 in tissues of H1299 cell xenograft tumors as described in E. All data are illustrated as mean ± SD and the asterisks show difference significant as *P < 0.05, **P < 0.01, ***P < 0.001 compared with the Control group or between the two groups shown by a horizontal line

To investigate whether TMS treatment could overcome resistance of NSCLC cells to gefitinib, we selected the gefitinib resistant H1299 and PC‐9/GR cells as examples and the cell proliferation, cell apoptosis levels and miR‐345/miR‐498 expression were analysed. To confirm whether TMS had a dose and time‐dependent effect on cell growth, a concentration gradient (0, 0.5, 5, 50, 500 μmol/L) was designed and the inhibition of cell proliferation was detected by a colorimetric MTT assay. Results showed that increasing TMS concentrations and action times both increased the inhibition rates of H1299 and PC‐9/GR cells, within a certain concentration range (Table 1). We selected 5 μmol/L TMS and 10 μmol/L gefitinib in subsequent experiments. When used alone, both TMS and gefitinib had good antitumour effects, which promote cell apoptosis with a combination of gefitinib and TMS, their pro‐apoptotic effects were more significant (Figure 2A). Xenograft tumors growth in athymic mice by H1299 cells subcutaneous injection, and when palpable tumours developed, these mice were treated with gefitinib or/and TMS by daily oral administration for about a month. The growth rates of tumour volume and weight were smaller in gefitinib and TMS groups than in the Control group indicating that gefitinib and TMS significantly inhibited tumour growth, but that the combined inhibition of gefitinib and TMS was more remarkable (Figure 2D‐F). Gefitinib showed a slight increase in the expression of miR‐345 and miR‐498 in H1299 and PC‐9/GR but also in xenograft tumours, while TMS significantly upregulated their levels and their increases were more observable when combined with gefitinib (Figure 2B,G). The detection of apoptotic markers in mRNA and protein expression levels also verified that TMS decreased gefitinib resistance of H1299 and PC‐9/GR cells by increasing the expression of caspase‐3 and suppressing the level of Bcl‐2/Bax (Figure 2B,C).

**Table 1:** TMS inhibited cell proliferation with a dose‐ and time‐dependent pattern

Cell lines	TMS (μmol/L)	Inhibitory rate (%)			*P*
		24 h	48 h	72 h	
PC‐9/GR	0	9.56 ± 1.25	15.26 ± 1.06	26.87 ± 2.14	<0.01^a^
	0.5	18.69 ± 1.36	25.48 ± 2.15	34.59 ± 1.78	<0.01^a^
	5	31.69 ± 1.89	36.78 ± 2.89	58.78 ± 2.01	<0.01^a^
	50	45.64 ± 2.06	56.78 ± 1.97	72.54 ± 3.25	<0.01^a^
	500	56.98 ± 3.16	71.87 ± 2.48	86.47 ± 0.98	<0.01^a^
	*P*	<0.01^b^	<0.01^b^	<0.01^b^	
H1299	0	7.56 ± 1.45	15.48 ± 2.01	31.89 ± 1.48	<0.01^a^
	0.5	14.69 ± 2.34	27.48 ± 2.57	40.69 ± 1.57	<0.01^a^
	5	24.56 ± 3.01	41.67 ± 0.89	56.98 ± 3.14	<0.01^a^
	50	46.78 ± 1.87	60.47 ± 2.87	76.98 ± 2.58	<0.01^a^
	500	72.15 ± 3.14	80.45 ± 2.78	91.34 ± 2.67	<0.01^a^
	*P*	<0.01^b^	<0.01^b^	<0.01^b^	

a *P* value in groups of different times.

b *P* value in groups of different concentrations.

### 3.3 MiR‐345 and miR‐498 inhibited H1299 and PC‐9/GR cell proliferation by inducing cell cycle arrest

**Figure 3:**
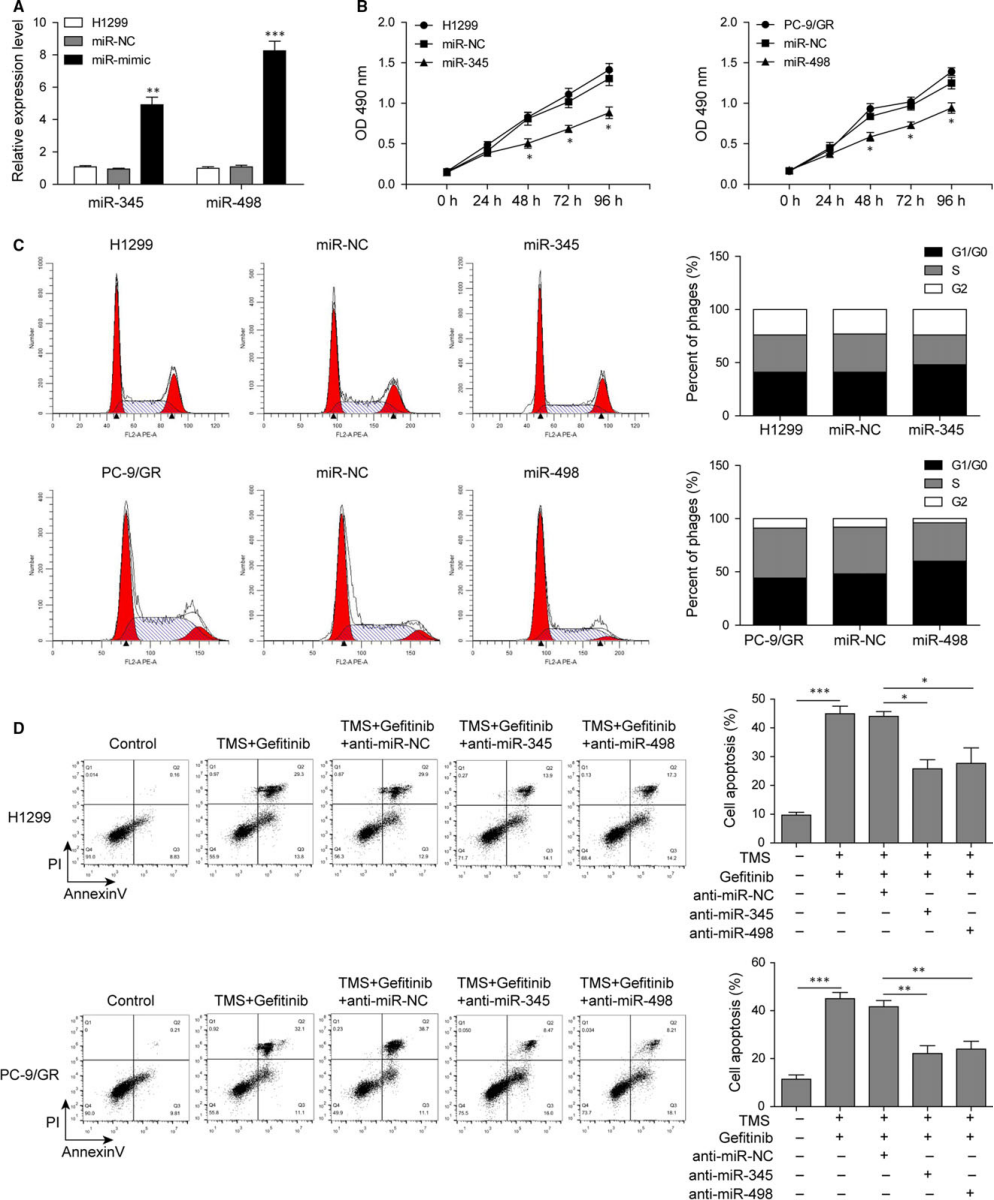
MiR‐345 and miR‐498 inhibited H1299 and PC‐9/GR cell proliferation by inducing cell cycle arrest. (A) Detection of miR‐345 and miR‐498 levels in H1299 cells after transfection with miR‐345 mimic, miR‐498 mimic or miR‐NC for 24 h by qRT‐PCR. (B) Cell proliferation rates of H1299 cells overexpressed with miR‐345 or PC‐9/GR cells overexpressed with miR‐498. (C) Cell cycle analysis of H1299 and PC‐9/GR cells treated with miR‐345 or miR‐498 by flow cytometry. Proportion of cells at different stages was analysed and displayed in the histogram. (D) Apoptosis analysis of H1299 and PC‐9/GR cells treated combined with TMS and gefitinib along with or without miR‐345/miR‐498 inhibitors by Annexin‐V/PE and flow cytometry. All data was illustrated as mean ± SD and the asterisks show significant difference as *P < 0.05, **P < 0.01, ***P < 0.001 compared with the miR‐NC group or between the two groups shown by a horizontal line

To investigate the effects of miR‐345 and miR‐498 on NSCLC, miRNA mimics were used to overexpress miR‐345 and miR‐498 in H1299 (Figure 3A) and PC‐9/GR and cell growth curve and cell cycle were analysed by MTT assay and flow cytometry. Results displayed that miR‐345 and miR‐498 overexpression inhibited H1299 and PC‐9/GR cell proliferation (Figure 3B) and induced cell cycle arrestin G1/G0 phage (Figure 3C) compared with blank control and miR‐NC groups.

In order to further confirm whether it was the upregulation of miR‐345 and miR‐498 by TMS to promote cell apoptosis, miRNA inhibitors were used to antagonize miR‐345/miR‐498 and flow cytometry analysis was performed. As shown in Figure 3D, inhibition of miR‐345 or miR‐498 weakened the effect of TMS and restored cellular resistance to gefitinib by decreasing apoptosis.

### 3.4 MiR‐345 and miR‐498 regulated the MAPK/c‐Fos and Akt/Bcl‐2 pathways by targeting MAPK1 and PIK3R1 respectively

**Figure 4:**
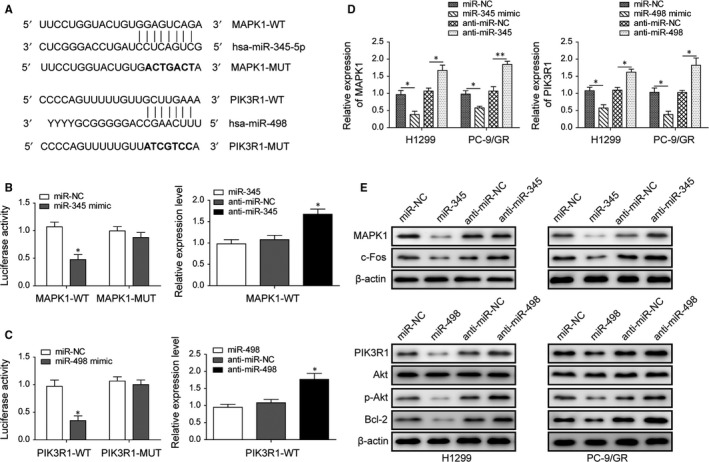
MiR‐345 and miR‐498 regulated the MAPK/c‐Fos and Akt/Bcl‐2 pathways by targeting MAPK1 and PIK3R1 respectively. (A) Schematic picture of the predicted interaction sites between miR‐345 and MAPK1‐3′UTR, miR‐498 and PIK3R1‐3′UTR, in addition to the designed mutations (in bold). (B) The dual‐luciferase reporter assay analysis in HEK‐293 cells co‐transfected with reporter plasmid inserted with wild‐type or mutated MAPK1‐3′UTR sequences and miR‐345 or miR‐NC. (C) The dual‐luciferase reporter assay analysis in HEK‐293 cells co‐transfected with reporter plasmid inserted with wild‐type or mutated PIK3R1‐3′UTR sequences and miR‐498 or miR‐NC. (D) The gene expression analysis of MAPK1 and PIK3R1 in H1299 and PC‐9/GR cells treated with miR‐345/miR‐498 mimics or inhibitors. (E) Immunoblotting analysis of MAPK1/PIK3R1 and their downstream proteins expression in H1299 and PC‐9/GR cells treated with miR‐345/miR‐498 mimics or inhibitors. All data are illustrated as mean ± SD and the asterisks show significant difference as *P < 0.05, **P < 0.01 compared with the miR‐NC/anti‐miR‐NC group or between two groups shown by a horizontal line

Software prediction by TargetScan, DIANA Tools and miRDB found MAPK1, a vital member of MAPK family, is a target of miR‐345 and PIK3R1, an essential component of the PI3K/Akt signalling pathway, is a target of miR‐498.^21^ The potential interaction sites of miRNA and the 3′‐UTR of its target genes were analysed using the RNAhybrid 2.2 software and mutations were introduced into these sites (in bold) to create an miRNA insensitive 3′‐UTR (Figure 4A). The dual‐luciferase reporter assays displayed that miR‐345 inhibited the luciferase activity with wild‐type MAPK1‐3′‐UTR (MAPK1‐WT) but had no significant effect on the luciferase activity with mutant MAPK1‐3′‐UTR (MAPK1‐MUT). Inhibition of miR‐345 enhanced the luciferase activity with wild‐type MAPK1‐3′‐UTR (Figure 4B). As expectedly, similar findings were also obtained on the interaction of miR‐498 between with the wild‐type PIK3R1‐3'UTR (PIK3R1‐WT) or the mutant PIK3R1‐3'UTR (PIK3R1‐MUT) (Figure 4C). The effects on expression of targets by miRNA were also verified at the transcription and protein levels. As shown in Figure 4D,E, miR‐345 and miR‐498 downregulated their targets expression both at mRNA and protein levels, while inhibition of miR‐345 or miR‐498 conversely upregulated these targets. MiR‐345 and miR‐498 also regulated the expressions of c‐Fos and Akt/Bcl‐2, which are downstream signalling molecules of MAPK and PI3K pathways.

### 3.5 TMS overcomes gefitinib resistance in H1299 and PC‐9/GR cells by inhibiting MAPK/c‐Fos and Akt/Bcl‐2 signalling pathways through miR‐345 and miR‐498

**Figure 5:**
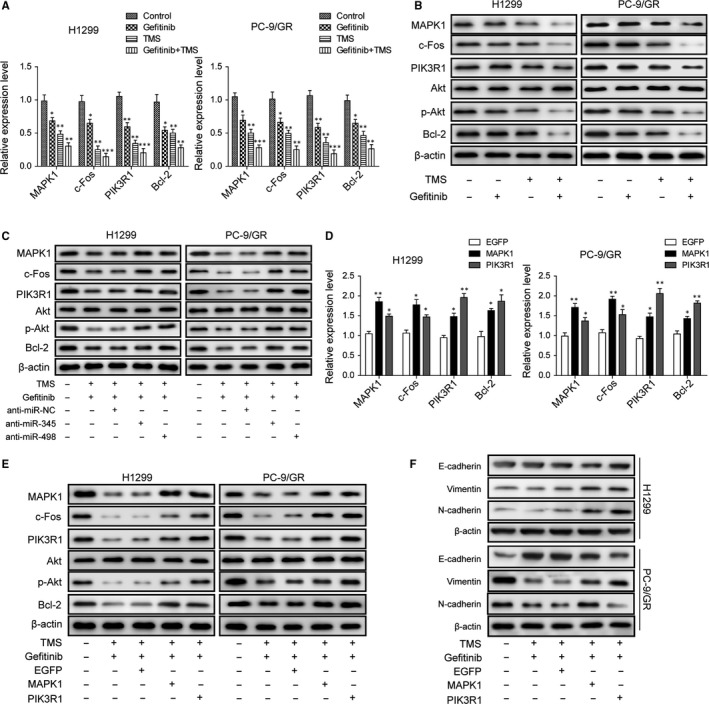
TMS overcome gefitinib‐resistance in H1299 and PC‐9/GR cells by inhibiting MAPK/c‐Fos and Akt/Bcl‐2 signalling pathway through miR‐345 and miR‐498. (A) qRT‐PCR analysis of the gene expression levels of MAPK1, c‐Fos, PIK3R1 and Bcl‐2 in H1299 and PC‐9/GR cells treated with or without TMS and gefitinib. (B) Immunoblotting analysis of MAPK1/PIK3R1 and their downstream proteins expression in H1299 and PC‐9/GR cells treated with or without TMS and gefitinib. (C) Immunoblotting analysis of MAPK1/PIK3R1 and their downstream protein expression in H1299 and PC‐9/GR cells treated combined with TMS and gefitinib along with or without miR‐345/miR‐498 inhibitors. (D) qRT‐PCR analysis of the mRNA levels of these genes, MAPK1, c‐Fos, PIK3R1 and Bcl‐2, after overexpression of MAPK1 and PIK3R1 in H1299 and PC‐9/GR cells. (E) Immunoblotting analysis of MAPK1/PIK3R1 and their downstream proteins expression in H1299 and PC‐9/GR cells treated combined with TMS and gefitinib after MAPK1 or PIK3R1 overexpression for 24 h. (F) Immunoblotting analysis of EMT marker expression in H1299 and PC‐9/GR cells described in E. All data are illustrated as mean ± SD and the asterisks show significant difference as *P < 0.05, **P < 0.01, ***P < 0.001 compared with the Control/GFP group

To confirm the effects of TMS combined with gefitinib on downstream signalling pathways, expression levels of key molecules were analysed by qRT‐PCR and western blot. With the elevation of miR‐345 or miR‐498 induced by TMS or gefitinib alone or combined use of the two, expression of their target and the downstream signalling molecules, such as MAPK1, c‐Fos, PI3K, phosph‐Akt and Bcl‐2 were decreased. Compared to the other three groups, every mRNA level and protein level, the targets expression levels in the TMS and gefitinib combination group were the lowest (Figure 5A,B) suggesting the inhibitory rate of TMS combined with gefitinib was greater and more significant. However, inhibition of miR‐345 or miR‐498 by miRNA antagonists neutralized the combined effects of TMS and gefitinib (Figure 5C). Overexpression of MAPK1 or PIK3R1 also played a similar role to reactivate the MAPK/c‐Fos and Akt/Bcl‐2 signalling pathways and promote cell growth and proliferation diminishing the antitumour and gefitinib sensitization of TMS (Figure 5D,E). Recent studies have indicated that, the PC‐9/GR cell subline achieves acquired resistance (AR) through the development of EMT. So, the expression of EMT markers after TMS treatment was analysed. Data indicated that TMS treatment significantly reduced EMT by upregulating E‐cadherin and downregulating vimentin and N‐cadherin in PC‐9/GR cells, but these effects were reversed by the overexpression of MAPK1 or PIK3R1 suggesting that the gefitinib‐AR of PC‐9/GR cells was reduced. However, these effects were relatively less dramatic in H1299 cells because of the weaker EMT in H1299 cells compared to PC‐9/GR cells (Figure 5F). All these results suggested that TMS overcame gefitinib resistance in H1299 and PC‐9/GR cells by inhibiting the MAPK/c‐Fos and Akt/Bcl‐2 signalling pathways.

### 3.6 TMS enhanced gefitinib chemosensitivity on tumour growth inhibition by suppressing the MAPK/Akt/Bcl‐2 pathway through miR‐345 and miR‐498

**Figure 6:**
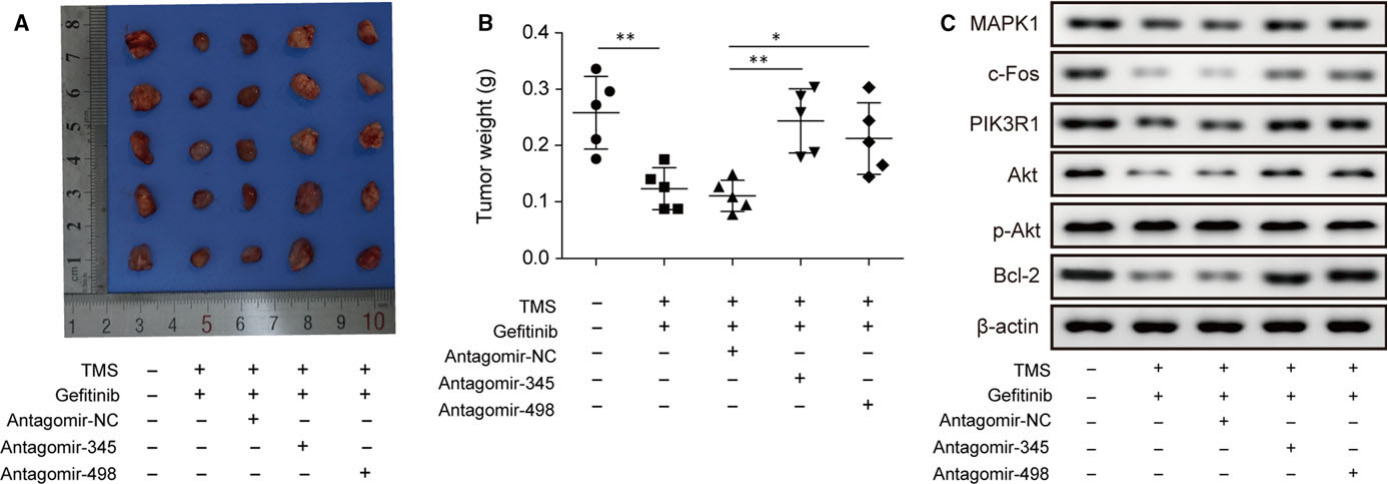
TMS enhanced gefitinib chemosensitivity on tumour growth inhibition by suppressing MAPK/Akt/Bcl‐2 pathway through miR‐345 and miR‐498. (A) Observation of xenograft tumours formed by H1299 cells in athymic mice combined‐treated orally with gefitinib/TMS and tumour injection with antogomiRNA oligonucleotides. (B) The tumour weights of H1299 cell xenografts as described in A. (C) Immunoblotting analysis of MAPK1/PIK3R1 and their downstream protein expression in xenograft tumour tissues as described in A. All data are illustrated as mean ± SD and the asterisks show significant difference as *P < 0.05, **P < 0.01

As described above, gefitinib and TMS significantly inhibited in tumour growth (Figure 2D,E) and the upregulation of miR‐345 and miR‐498 may be the cause (Figure 2F). For further verification, antagomir‐345 and antagomir‐498 were used to block their upregulation and the results revealed that the tumour growth inhibition of gefitinib/TMS was abolished by antagomir‐345 and antagomir‐498, and the tumour volume and weight increased in the antagomir‐345/antagomir‐498 groups compared to the antagomir‐NC group (Figure 6A,B). Similarly, the expression levels of the targets and downstream signalling molecules of miR‐345 and miR‐498 were also increased because of derepression by reduced miR‐345 and miR‐498 expression (Figure 6C). These findings further demonstrated in vivo that TMS enhanced the chemosensitivity of gefitinib on tumour growth inhibition by suppressing the MAPK/Akt/Bcl‐2 pathway through miR‐345 and miR‐498.

## 4 DISCUSSION

Gefitinib is a commonly used EGFR‐TKI drug for NSCLC treatment, with characteristics of strong specificity, good tolerance and few adverse reactions. However, acquired drug resistance is the main reason for the failure of clinical gefitinib treatment becoming an urgent problem to NSCLC therapy and study.^2^

At present, the sensitivity and resistance of chemotherapy drugs are related to apoptosis, a well‐organised process of cell death pre‐programmed inside the cell. Studies have shown that many anti‐tumour drugs can induce tumour cell apoptosis, such as alkylating agents and cisplatin.^22^ However, tumour cells can avoid apoptotic damage by improving the threshold of cell apoptosis or reducing cell susceptibility to apoptosis‐inducing factors leading to tumour drug resistance.^23^ Gefitinib promotes tumour cells apoptosis by competitive binding with ATP in the tyrosine kinase domain of EGFR (EGFR‐TKD) and specifically inhibiting EGFR tyrosine kinase activity.^24^ Gefitinib resistant tumour cells appear to inhibit apoptosis and combination treatment with other drugs may reverse the resistance to gefitinib by enhancing apoptosis levels.

Resveratrol, a natural polyphenols has been reported to possess anticancer properties in a wide variety of tumour cell types including breast, prostate, stomach, colon, pancreas and thyroid cancers.^20^, ^21^ It can suppress proliferation, invasion and apoptosis by regulating NF‐κB and activator protein‐1 activities.^18^, ^19^ One of the resveratrol derivatives TMS was found to be more potent than resveratrol as an anticancer agent.^9^, ^11^ In this study, we evaluated the anticancer effects in vitro and in vivo. To uncover the underlying molecular mechanisms of TMS, we also studied the cell cycle and gene expression in gefitinib resistant NSCLC cells and subcutaneous xenograft tumours. Results showed that TMS decreased resistance to gefitinib by promoting apoptosis in H1299 and PC‐9/GR cells and inhibiting the tumour growth in athymic mice.

Currently, a number of miRNAs have been described which may have a specific role in lung cancer pathogenesis, biological and clinical disease behaviour as well as in modulating response to anticancer treatments, particularly EGFR‐TKIs.^23^, ^24^, ^25^, ^26^ MiR‐345 and miR‐498 were downregulated in NSCLC patients and cell lines, closely correlating with the tumour progression and poor prognosis.^16^, ^17^ Our data found that there was a lower expression of miR‐345 and miR‐498 in gefitinib resistant cells than gefitinib sensitive cells. Furthermore, overexpression of miR‐345 and miR‐498 inhibited H1299 and PC‐9/GR cell proliferation by inducing cell cycle arrest. Then, we further speculated whether TMS regulated EGFR‐TKI resistance by low‐level miR‐345 and miR‐498. The results verified that TMS treatment upregulated miR‐345 and miR‐498 expression in H1299 and PC‐9/GR cells inhibiting proliferation and inducing apoptosis. Similar changes were detected in xenograft tumour tissues.

More researches targeting the downstream signalling pathways further supported our findings. The over‐activation of the PI3K/Akt and MAPK/ERK signalling pathways is common in tumours and also associated with malignant transformation and drug resistance.^25^, ^26^ Blocking these pathways by MAPK/PI3K inhibitors can inhibit cell proliferation of NSCLC.^20^ EGFR‐TKIs block the PI3K/Akt and MAPK/ERK signalling pathways downstream of EGFR in highly sensitive NSCLC cell lines.^25^, ^27^, ^28^ However, in resistant cell lines, EGFR inhibition by EGFR‐TKIs could not lead to apoptosis of cancer cells.^25^, ^29^, ^30^ Therefore, inhibition of PI3K/Akt and MAPK signalling pathways through other ways may be an effective strategy for improving EGFR‐TKI resistance. In our study, we have verified that MAPK1 and PIK3R1 are the direct targets of miR‐345 and miR‐498 respectively. And the increase of miR‐345 and miR‐498 induced by TMS suppressed MAPK1 and PIK3R1 protein expression leading to the blocking of MAPK and PI3K/Akt signalling pathways, cell proliferation inhibition and apoptosis promotion. Suppressing the increase of miR‐345 and miR‐498 induced by TMS or overexpression of MAPK1/PIK3R1, both neutralized the effects of TMS treatment on the MAPK and PI3K/Akt signalling pathways in vitro and in vivo.

The PC‐9/GR cell is a gefitinib‐acquired resistant cell subline of PC‐9 cells, which has an EGFR‐activating mutation, which is a 15‐bp deletion in the EGFR exon 19 and is initially sensitive to gefitinib.^18^ The gefitinib resistance mechanism is very complex, may relate to secondary mutation of EGFR, c‐Met amplification, compensatory signal establishment and the change in regulatory factors expression and the tumour microenvironment transformation. Cheng et al detected that there was a T790M mutation which may be the cause of gefitinib‐AR for PC‐9/GR cells, although it occurs less frequently.^18^ Recent studies have also indicated that there was not a T790M mutation in the PC‐9/GR cell subline; however, it achieved AR through development of EMT. The expression of E‐cadherin decreased but vimentin and N‐cadherin increased in PC‐9/GR cells compared to the PC‐9 cells indicating that PC‐9/GR cells acquired the EMT phenotype.^31^ As we know, MAPK and PI3K signalling pathways are involved in inducing tumour EMT and play roles in drug resistance.^32^, ^33^ In our study, we have verified that TMS increased the sensitivity to gefitinib by reduced EMT by upregulating E‐cadherin and downregulating vimentin and N‐cadherin in PC‐9/GR cells after suppressing MAPK and PI3K/AKT signalling pathways by upregulating miR‐345/miR‐498. But these effects were reversed by the overexpression of MAPK1 or PIK3R1, further suggesting that the gefitinib‐AR of PC‐9/GR cells was reduced by TMS. However, these effects were relatively less dramatic in H1299 cells because of the weaker EMT in H1299 cells, which were primarily resistant to gefitinib caused by TP53 deficiency and NRAS mutation.^34^

In conclusion, we have demonstrated that the resveratrol derivative TMS could reduce gefitinib resistance in NSCLC cells and xenograft tumour tissues by blocking the PI3K/Akt and MAPK pathways by upregulating miR‐345 and 498. Our data would lay the theoretical basis for the future study of TMS for the treatment of EGFR‐TKI resistance in NSCLCs.

## CONFLICT OF INTEREST

All authors declare no conflicts of interest.
